# Danger of Injections of Perchloride of Iron in Sanguineous Tumors

**Published:** 1869-11

**Authors:** 


### Danger of Injections of Perchloride of Iron in Sanguineous Tumors.

A child ten weeks old having three sanguineous tumors respectively on left cheek, left leg, and right lumbar region, was brought to Professor Sautesson, of Stockholm. Whilst in the country the child had been for two weeks in charge of a physician, who was prevented from vacinating the tumor on the face by want of virus, and instead applied collodion, bnt this produced no change.

The tumors were beneath the skin and evidently still growing. That on the face, the one of greatest importance, was an inch in diameter, and occupied more than half the thickness of the cheek, being situated midway on a line drawn from the ala-nasi to the lobule of the ear. Vaccination not promising any hope of success, and such procedures as excision, ligation and cauterization presenting serious objections, the choice was limited to acupuncture by galvanic needle, and injection of a coagulating liquid. The latter was preferred by the Professor, and immediately, with the assistance of Prof. Abelin and Dr. Schlerg, the injection of a liquid composed of ferri perchloridi six parts, and alcohol one part, was commenced. The injection was made with a subcutaneous glass syring containiug eight or ten drops of the mixture. The capillary tube was first introduced and directed vertically across the tumor, towards its centre, and only one-half of its contents injected, it was then withdrawn and re-introduced, horizontally, the point being directed deeper in the tumor. Before the second injection was completed, the operator was forced to withdraw the syringe on account of the appearance of threatening symptoms in the child. Death took place in about two hours.

Autopsy on the day following death, revealed the following symptoms: The tumor was much lessened in size, its tissue from being spongy, had become firm and solid from coagulation of the blood.

The surrounding veins (facial and its ramifications) were empty. The external and internal jugulars contained no clots in the superior portion, but as they approached the chest, the contained blood was generally clotted. The clots became more and more firm in the subclavian, the superior vena cava and in the right cavities of the heart. These were literally distended with coagulated blood. The left au ricle contained a small clot, the left ventricle was empty. It is probable that in introducing the tube the second time, one of the veins (perhaps a branch of the facial) was penetrated. The history of this case, and its unfortunate termination, suggests as a precautionary measure against a similar occurence, pressure between the tumor and the heart when performing the injection.—*(Monatssh) bh. South—Union Medical, May* 15*th*, 1869.

